# Parental perceptions of an oral health promotion program in early childhood education and care settings: A qualitative study

**DOI:** 10.1111/jphd.12641

**Published:** 2024-08-29

**Authors:** Lauren Suzanne Heller, Rashmi Pithavadian, Sowbhagya Micheal, Navira Chandio, Prathyusha Sanagavarapu, Jinal Parmar, Susan Cartwright, Linda Slack‐Smith, Amit Arora

**Affiliations:** ^1^ School of Health Sciences Western Sydney University Campbelltown New South Wales Australia; ^2^ Health Equity Laboratory Campbelltown New South Wales Australia; ^3^ School of Medicine Western Sydney University Campbelltown New South Wales Australia; ^4^ Translational Health Research Institute, Western Sydney University Campbelltown New South Wales Australia; ^5^ School of Education Western Sydney University Bankstown New South Wales Australia; ^6^ Colgate‐Palmolive Company Sydney New South Wales Australia; ^7^ Paediatric epidemiology University of Western Australia Perth WA Australia; ^8^ Discipline of Child and Adolescent Health, Sydney Medical School, Faculty of Medicine and Health The University of Sydney Westmead New South Wales Australia; ^9^ Oral Health Services, Sydney Local Health District and Sydney Dental Hospital, NSW Health Surry Hills New South Wales Australia

**Keywords:** early childhood caries, early childhood education and care setting, oral health promotion, preschool, qualitative, toothbrushing

## Abstract

**Objectives:**

Early Childhood Caries is a global health problem. The Bright Smiles Bright Futures (BSBF) program seeks to equip educators, children, and parents with skills and knowledge about oral health promotion habits early in life. The aim of this study was to examine parental perceptions of the BSBF program and identify key facilitators and barriers for its implementation.

**Methods:**

Twelve mothers of children who participated in the BSBF program in five Early Childhood Education and Care (ECEC) settings in NSW, Australia were recruited in this qualitative study. Data were collected via focus groups and interviews, transcribed verbatim and coded to categorize for inductive thematic analysis.

**Results:**

Five major themes emerged: Promoters of the BSBF oral health program, barriers to the BSBF oral health program implementation and participation, online resources, impact of the BSBF oral health program, and strategies for enhancing the BSBF oral health program. While participants reported that the program encouraged their children's toothbrushing, they found issues with the program's materials, ECEC center attendance, and communication about the oral health program with their children. The program improved message retention, attitudes, routines, and family perceptions toward oral health. Participants recommended oral health literacy, changed delivery formats, increased dental access, and inclusion of interactive elements to enhance the program.

**Conclusions:**

The findings from this study provide insight to improve parents' experiences and engagement in oral health promotion. This can help to raise awareness of the importance of child oral health among policymakers, healthcare professionals, and the public to inform public health policy discussions.

## INTRODUCTION

Early Childhood Caries (ECC) is a severe form of tooth decay that affects children under the age of 6 years [1]. ECC affects approximately 48% of preschool children worldwide [2] and The Global Burden of Disease Study estimated approximately 530 million children experience dental caries in their primary (baby) teeth [3]. The most recent Australian National Child Oral Health Study (2012–2014) reported that approximately over 34% of children experienced tooth decay in primary teeth at 5–6 years [4]. ECC impacts a child's oral health and has far‐reaching consequences on their overall quality of life by influencing their self‐confidence, socialization, learning abilities, nutritional intake, and growth [5]. Between 2020 and 2022, in Australia, the rate of preventable hospitalizations for dental conditions was highest (nearly 11 in every 1000 children in the population) in children [6]. This highlights the need for more targeted preventive strategies [6] to prevent dental caries in young children with a multidimensional care‐model that encompasses a holistic approach to address ECC from perspectives of children, family, and the community [7]. Despite the complex etiology of ECC, it is critical to note that ECC is preventable [8]. Oral health professionals recommend regular toothbrushing, low‐sugar diet, regular dental visits, and community water fluoridation as preventative strategies for ECC [9].

The World Health Organization (WHO) introduced the Global School Health Initiative in 1995 to mobilize and strengthen health promotion and education activities at local, national, regional, and global levels [10]. In 2003, the WHO highlighted the need to capitalizing on schools as a setting for oral health promotion [11, 12]. Since then, there have been a number of oral health promotion and education programs being implemented in educational settings as they play a significant role to promote oral health literacy among children and parents [13]. Statewide programs such as “Empower” and “Growing Healthy Smiles” have been introduced in the United States [14, 15]. Oral health promotion programs, including “Clean Teeth, Wicked Smiles” and “Happy Teeth: Resource Kit”, have also been implemented in Australian schools [13]. However, there is limited knowledge on parents' experiences and perceptions of oral health interventions in Early Childhood Education and Care (ECEC) settings [16, 17]. Such insights are crucial for formulating effective and sustainable pediatric oral health strategies.

Parental roles, perceptions, and views on oral health is crucial for pediatric oral health. Gläser‐Ammann et al. [18] found that 72% of parents recognized the significance of oral health programs implemented in school. However, Chandio et al. [19] highlighted that parental involvement and responsiveness to these programs could be hindered by inadequate engagement, insufficient program communication, and misinformation. It is imperative to note that an increased incidence of ECC may be attributed to a combination of factors, including social determinants and competing time challenges, which may hinder parents' abilities to maintain consistent oral health routines and enhance their oral health literacy [20, 21]. Despite the existence of oral health promotion programs, interventions are often not implemented before the onset of ECC in the early childhood years [22]. Therefore, more focus is needed on both oral health programs in early childhood years and parents' perceptions of these programs to reduce the burden of ECC and promote oral health.

The Bright Smiles Bright Futures (BSBF) program was developed by Colgate‐Palmolive Company (New York, NY, USA) in the United States in 1991 [23]. The BSBF has provided free educational materials to over a billion children in 80 countries and benefitted approximately 8.9 million children in Australia [23]. The BSBF program provides educators with teaching materials, children with take‐home brochures for toothbrushing, a chart for tracking brushing, and online resources for children and parents on the BSBF website. Our previous research assessed the children's perceptions of the BSBF program in NSW, Australia [24]. However, there is a lack of research focused on parental perspectives regarding the execution of early childhood oral health programs in an Australian context. To address the research gap, this study aims to examine parental perceptions of the oral health BSBF program in Australia and identify key facilitators and barriers for its implementation during early childhood years.

## METHODS

### Research design

A qualitative research design was employed to capture parents' rich viewpoints on the oral health program [25]. This helped to identify parents' collective experiences and perspectives, as well as any unique or unconsidered insights in the overlooked area of oral health practices among preschool‐aged children and their families [26, 27]. Semi‐structured interviews and focus groups were used as qualitative data collection methods, which provided open‐ended questions for participants to respond with detailed perspectives [25]. Such flexibility allowed the emergence of unconsidered perspectives [26]. By embracing this methodology, our study aimed to organically distil the varied parental viewpoints elicited post‐implementation of the BSBF oral health project, allowing for further exploration of their experiences and perceptions [26]. The consolidated criteria for reporting qualitative research (COREQ) were followed [27]. The completed COREQ checklist is provided in Table S1.

### Ethical considerations

This study was approved by the Human Research Ethics Committee at Western Sydney University (approval number H14372). Pseudonyms were used in the data analysis and reporting of findings for participant confidentiality.

### Recruitment

Purposive sampling was employed for data collection [28]. Participants had to meet the following eligibility criteria: (i) have a child that was enrolled in the selected ECEC center associated with the NSW Department of Education that implemented the BSBF program, (ii) able to communicate in English and could understand written and spoken English, (iii) aged 18 years or above at the time of enrolment. The director and teachers of the selected ECEC centers, which implemented the BSBF program were contacted to help recruit parents who met the eligibility criteria as participants. Parents at selected ECEC center were invited to participate in the study and were sent a take‐home information pack consisting of a participant information sheet and a consent form. This led to 12 mothers of children who participated in the BSBF program to be recruited in this study after they provided written informed consent. The geographic locations of the ECEC centers included both metropolitan and rural locations of NSW in Australia, which included Springwood, Cronulla, Peakhurst, Bathurst, and Cessnock. The diversity in geographic location increased representation in the sample and offered insight into parents' unique perspectives of oral health. To ensure a comprehensive understanding of the geographic, demographic, and socioeconomic profiles of the study areas, we used the Modified Monash Model (MMM) and Socio‐Economic Indexes for Areas (SEIFA). The metropolitan areas of Springwood, Cronulla, and Peakhurst in NSW are classified as MMM 1, indicating metropolitan regions with high population density and greater availability of healthcare facilities [29]. SEIFA rankings show that Cronulla and Peakhurst have relatively high socio‐economic status, while Springwood is in the middle range [30]. In contrast, the rural towns of Bathurst and Cessnock are classified as MMM 3, representing large rural towns with distinct challenges in delivering early childhood oral health programs, including limited access to healthcare services and lower socio‐economic status according to SEIFA rankings [30].

### Data collection

After providing written informed consent, the primary researcher (LSH) emailed or phoned the 12 mothers to schedule them into a focus group or one‐on‐one interview via Zoom videoconferencing, which were recorded. Focus groups and interviews were facilitated by LSH and RP between November 2022 and April 2023. A semi‐structured interview/focus group guide of 12 questions presented in Table 1 was used to allow parents to provide feedback on the oral health.

**TABLE 1:** Semi‐structured focus group/interview guide for parents.

What were your experiences with the oral health promotion program?
2 What did your child share about their experiences with the oral health in‐school activities?
3 Did your child tell you about any messages they learned from the oral health lessons in school?
If they did, what were these messages?
4 What changes, if any, did you see in your child's, or your family's, oral health routine because of the program?
5 There was a digital component to the oral health program for parents which was the website (screenshare to display website). Were you able to access the website?
6 If you were, what are your thoughts about the website?
7 What do you think are challenges in promoting oral health with your child and family?
8 What are some ways to improve parents' understanding of oral health?
9 How can oral health programs support you and your children to discuss oral health?
10 What are strategies to ensure children brush their teeth twice a day?
11 How do you think we can ensure that the importance of oral health is emphasized throughout school and the general community?
12 Is there anything else you want to say, share, or comment about regarding the oral health program?

In total, there were three focus groups with two participants in each and six one‐on‐one interviews. Each focus group/interview lasted between 20 and 40 min. Unlike in‐person focus groups, online focus groups are better suited to a smaller number of participants to allow for easier interaction and visualization of all participants online [31]. Each participant was reimbursed for their time with a $50 gift voucher. LSH and RP developed field notes after each interview or focus group.

### Data analysis

The audio recordings from the interviews and focus groups were transcribed using the online transcription program Trint. These transcripts were then verified against the audio‐recording. The authors applied a naturalized approach to transcription by retaining participants' stutters, word fillers, and repetition to avoid altering their expression of perspectives [32]. LSH and RP read their field notes and transcripts three times for data immersion. This helped them both to devise an initial draft‐coding frame for inductive thematic analysis focused on participants' experiences and perspectives of the program. Quirkos software was then used to undertake line‐by‐line coding of the transcripts according to the draft‐coding frame. Quirkos software allowed LSH and RP to visually revise codes by deleting, merging, or renaming them. AA and SM reviewed codes and made suggestions for revisions. LSH and RP discussed coding revisions until consensus was reached to uphold qualitative trustworthiness and rigor. Data adequacy was reached when no new codes could be devised within the scope of the study [33]. This aligns with Hennink & Kaiser's [34] recommendation that 9–17 interviews and/or 4–8 focus group discussions typically reach data adequacy. The final codes are presented as themes in the results.

### Methodological rigor

To enhance study credibility, qualified researchers with previous qualitative research experience conducted interviews and focus groups through Zoom videoconferencing. Verbatim transcription was undertaken through cross‐checking each document twice. Codes were developed by LSH and RP and reviewed by AA and SM to uphold credibility and confirmability. LSH and RP employed negative case analysis to strengthen the rigor [35]. The study results included methodological information to replicate the study for dependability and transferability. Participant quotes are provided to demonstrate that findings were data‐driven for robust qualitative research as part of confirmability [36].

### Researcher positionality

The ways that researcher positionality influenced the research process of the study was considered [37, 38]. Therefore, reflexivity was employed to acknowledge the potential impact of the researchers' positions on the study. Except for the fourth and seventh authors, all other authors are public health researchers. The fourth author specializes in early childhood education. While the sixth author is qualified in public health management, the seventh author is a scientific affairs and public health manager at Colgate‐Palmolive but was not involved in data collection to manage potential conflict of interest. The last author also has qualifications in dentistry. Therefore, the varied positionalities of the authors supported analysis and interpretation of the interdisciplinary research focus in oral health, public health promotion, and early childhood education. The mixed researcher positionalities helped to balance analysis and inform critical discussion. Steps taken to ensure impartiality involved independent participant recruitment, maintaining a nonjudgmental approach during interviews/focus groups, and adopting an iterative data analysis method to ensure rigor [37, 38].

## RESULTS

The sample of 12 mothers had an average age of 36.5 years. Participants' children were aged 2–5 years old, with an average age of 4 years old, at the time of the focus groups and interviews. Seven participants had male children. The full demographic information of the sample of the 12 mothers and their children is displayed in Table 2.

**TABLE 2:** Demographics of study participants.

	*n* (%)	M	SD
Mother's age (years)		36.50	3.61
Child's age (months)		50.03	12.36
Child's sex			
Male	07 (58.3%)		
Female	05 (41.7%)		
Number of children in household			
One	01 (8.3%)		
Two or more	11 (91.7%)		
Mother's education			
Advanced diploma	01 (8.3%)		
High School diploma	01 (8.3%)		
Undergraduate degree	08 (66.7%)		
Postgraduate degree	01 (8.3%)		
Doctoral or beyond	01 (8.3%)		
Mother's geographical location			
Rural NSW	04 (33.3%)		
Metropolitan NSW	08 (66.7%)		
M‐mean, SD‐standard deviation of sample			

Five themes and relevant subthemes were identified from thematic analysis. The five themes were: Promoters of the BSBF oral health program, barriers to the BSBF oral health program implementation and participation, online resources, impact of the BSBF oral health program, and strategies for enhancing the BSBF oral health program. The themes and subthemes are summarized in Table 3.

**TABLE 3:** Themes and subthemes.

Themes	Subthemes
Theme 1: Positive behaviors promoted by the BSBF oral health program	Encouraging toothbrushing
	Helpful instructions for toothbrushing
Theme 2: Barriers to the BSBF oral health program implementation	Program material issues
	ECE center attendance
	Child and parent communication about the program
Theme 3: Online resources	Social media promotion
	Graphics, colors, and videos on BSBF website
	Feedback to redevelop BSBF website
Theme 4: Impact of the oral health program	Retaining key oral health messages
	Attitude towards toothbrushing
	Oral health routine
	Oral health promotion in the family
Theme 5: Strategies to enhance the BSBF oral health programs	Increase dental health literacy on sugary foods and drinks intake
	Format of message delivery
	Increase access to oral healthcare
	Mobile phone applications

### Promoters of the BSBF oral health program

#### Encouraging toothbrushing

Mothers mentioned the importance of encouraging good oral hygiene practices, specifically toothbrushing and flossing, as facilitators for their child's oral health. One mother explained that: “Clearly the messaging, that messaging gets through because she, yeah does, every evening remind me to brush her tongue because the‐, and like reminds me to brush top and bottom inside outside and her tongue.” Maya, 37 years old, rural NSW parent.

Maya's comment reflected parents' views of how the program encouraged toothbrushing in the family. Parents also highlighted how their child's participation in the program led to their understanding of the importance of flossing. One parent described how:

… *mostly I think for children like having that chart and just that kind of constant checklist that they can have a bit more independence with the activity*, *I think is is really good*. *And I know that like he*'*s learned through school*. Abigail, 39 years old, metropolitan NSW parent.

Abigail's comments reflected several parents' sentiments that having a chart or checklist help their child keep track of their oral hygiene practices, and that their children learned about positive oral hygiene messages through school.

#### Helpful instructions for toothbrushing

Mothers stated that the program provided helpful instructions to encourage more toothbrushing with their children. A mother described that: “Ben definitely shared more of a cleaning a full mouth. And which part is clean and how long he spent and, he's very much about are they sparkling, are they clean?” Emma, 44 years old, metropolitan NSW parent.

This highlighted several parents' view that their children learned more information on how to thoroughly clean their teeth to improve their oral health routine at home.

Mothers praised the program brochure's use of graphic formats to present helpful instructions. One mother stated how the graphic, step‐by‐step toothbrushing instructions within the brochure aided in her child's at‐home oral health routine:

*There was one that had brushing better in the steps with the photos*. *I think* … *for me as well*, *was really great*. *Like we*'*re always trying to explain it to him and* … *so having that illustrated so he can understand now that he*'*s brushing his own teeth and we still help him if we notice he*'*s maybe lazy*. *But I think having that*, *that he can kind of clearly understand as well was really cool*. Abigail, 39 years old, metropolitan NSW parent.

Abigail's statement captured other parents' comments, which highlighted the significance of using graphic formats to make instructions more engaging for children within oral hygiene within programs. Parents noted that this can aid in children's overall understanding of oral health.

### Barriers to the BSBF oral health program implementation and participation

#### Program material issues

Mothers referred to the challenges of managing the volume of paperwork and information that they receive from their child and their ECEC setting. Within this oral health program, a brochure was sent home with each child from their respective teacher. This brochure was often misplaced or not brought home by the child. One mother noted that the brochure is another piece of paper that can get lost among the other materials that come home: “Oh yeah, that's the thing that like we get a million letters from school and preschool and like there's just a lot of stuff coming home so it can get a bit lost in the chaos.”*—*Hannah, 36 years old, metropolitan NSW parent.

Hannah's acknowledgement that there is a lot of paperwork that comes home from school and preschool encapsulated many parents' comments that this can make it difficult to keep track of ECEC communication.

There may be limited engagement or follow‐up from the schools to the parents, which can impact the effectiveness of the project as another parent articulated:

*We really just heard about it through our daughter who came home and talked about the people who showed up that gave her stickers*, *gave her toothbrush*, *things like that really is the basic interaction that we had with it*. Elizabeth, 37 years old, metropolitan NSW parent.

Several parents noted, similar to Elizabeth, that their interaction with the program was limited to their child coming home and informing them about the people who came to the ECEC and the items that were given out to promote oral health.

#### ECEC center attendance

Children's attendance to their ECEC centers did not always align with when the oral health program ran. Children who were absent and missed oral health program lessons at their ECEC were unable to fully participate in the program.

One mother asked: “Was it a program that ran on every day? Like he doesn't attend every day.” Abigail, 39 years old, metropolitan NSW parent.

Similar to Abigail, other mothers also wondered whether the program ran every day, which implied that they did not have a clear understanding of the program's scheduling and duration.

Another mother commented that her child only attended the ECEC center certain days such as: “He goes this year, Monday–Wednesday. Last year he was Monday and Tuesday.” Amelia, 29 years old, rural NSW parent.

Amelia's response that the days her child attended ECEC center changed every year indicated that there may be variability in the program's scheduling from year to year or even among students.

#### Child and parent communication about the program

Parents indicated that they face challenges to understand what their children learned from the in‐school oral health promotion program. Ella stated that:

*If you guys gave me questions that would probably be helpful*. *Like yeah like to guide*, *guide the conversation or like kind of where we should be*, *what we should be talking about*. *Like*, *you know*, *whether it*'*s in terms of food or how to brush*. *Just like a list of questions would help*. Ella, 34 years old, metropolitan NSW parent.

Other mothers like Ella suggested that having a list of questions to guide the conversation of oral health promotion with their children would be helpful.

Mothers also described how difficult it is to communicate about oral health with their children:

*Well*, *I think it*'*s tricky*, *particularly this age group with pre‐schoolers that we just kind of get told*, *whatever they tell us*. *And like you can have a quick look at the brochure*, *but we don*'*t like, we are a bit blind as to what actually they*'*ve been taught and that sort of stuff. So I guess the only challenge there is like*, *I don*'*t really know what*'*s going on other than whatever gets given to me because you*'*re not there and they can*'*t explain it*. Hannah, 36 years old, metropolitan NSW parent.

Hannah's comment reflected other parents' views that it is difficult to communicate effectively about oral health with children at such a young age. They added that it can be challenging to know what their child has learned in school about oral health since they are not present.

### Online resources

#### Social media promotion

Parents wanted to receive oral health material via social media outlets in addition to the take‐home brochure and the online website. One mother stated that:

*Maybe if the preschool had sent it out*, *like on their Facebook page*, *a little like we got like internal things*. *To be fair*, *I probably wouldn*'*t have got around to it anyway*, *but*. *Yeah*, *I*'*d*‐, *I think we all know how to access*, *and it*'*s just whether you have the time*. Elizabeth, 37 years old, metropolitan NSW parent.

Elizabeth's comment echoed other parents' feedback that they want to receive oral health project information through social media platforms, such as Facebook, because they are more likely to access it during spare time.

Another mother revealed that: “… when you're on social media, when you've got all the time, so you're probably more likely to open it at the moment. Rather than seeing it and thinking oh yeah I should look at that and kind of get it.” – Emma, 44 years old, metropolitan NSW parent.

Other parents, like Emma, emphasized the need for social media links within educational material to deliver accessible and time efficient materials to parents.

#### Graphics, colors, videos on the BSBF website

Parents commented on the graphics, colors, and videos embedded within the website to engage their children in oral health. A mother noted:

*I had a look at some of the activities*. *And my daughter seemed kind of interested*, *she liked the maze one*, *looking for the what*'*s good and what*'*s bad*, *like lollies and veggies*, *whatever*. *And she*, *she liked the cartoons*. *So*, *the interactive stuff*. *She liked the cartoons themselves*. Hannah, 36 years old, metropolitan NSW parent.

Similar to Hannah, other parents highlighted how interactive activities and cartoons on the oral health promotion website were appealing to their children to engage them with the content.

Another mother explained that she appreciated the non‐screen‐oriented resources that were presented on the website:

*So having resources that are not videos oriented*. *I think is good*. *And very often a lot of those kind of educational programs like*, *you know*, *the Wiggles*, *toothbrushing song*, *all that kind of stuff*, *it*'*s very much focused on having a TV or a screen*. *And I just think we have so much screen focus that it*'*s fantastic to see something that*'*s got options that are not screen focused*, *like stuff that we can print ou*t. Maya, 39 years old, rural NSW parent.

Maya's comment reflected some parents view that downloadable printout material on the website can offer options to provide educational resources for parents who may not feel comfortable increasing much screen‐time with their children.

#### Feedback to redevelop the BSBF website

Mothers highlighted issues to access the BSBF website. After attempting to access the online resource, one mother mentioned that:

*Well*, *first thing was when I clicked on for parents*, *I got*, ‘*sorry no results were found*’. *That was kind of the first thing under the role when I clicked for parents. That*'*s what*. *So that didn*'*t help*. *Kind of navigating*, *I guess the website*. Ella, 34 years old, metropolitan NSW parent.

The difficulty Ella mentioned highlights the need to ensure that parents can easily access online resources within oral health programs to best promote usage and accessibility.

Another mother reported difficulty finding relevant information for parents on the website:

*I actually had a look at it yesterday and I didn*'*t think it was very user friendly*. *And it*'*s funny because I*'*m actually a user researcher as a background*, *so I test these kind of websites and I was looking for some sort of fact sheet*. *But oh*—*I was just*, *for me*, *I got very distracted*. *Actually*, *with all the cartoons everywhere I thought it was*, *I thought I*'*d be looking for like through the website*, *just sort of basic instructions on what to do with the kids and stuff like that*. *But it was sort of very*, *I was heavily taken aback by all the images*. Katie, 35 years old, metropolitan NSW parent.

A few other parents, like Katie, found the website to be distracting, too visually stimulating, and not user‐friendly. They were seeking useful resources and more‐practical information and instructions to engage their children with oral health, which they felt the website lacked.

### Impact of the BSBF oral health program

#### Retaining key oral health messages

Mothers' testimonials highlighted many messages their children retained from the oral health program. One mother explained:

*And every time we go*, *and he has something sweet*, *he*'*s like*, ‘*Oh*, *we can*'*t eat too much because then we*'*ll have to go to the dentist*.’ *And actually*, *he has a dentist appointment scheduled like next week*. *And*, *and then he*'*s always saying*, ‘*oh well*, *we have to use the drill*’. *And I*'*m like*, ‘*Well*, *only if you keep eating lollies*, *you know*? Katie, 35 years old, metropolitan NSW parent.

Parents, like Katie, noted how the oral health program improved their child's awareness of the connection between sugar consumption and dental health, and the potential consequences of not taking care of their teeth.

Another mother mentioned the impact of the program on improving her child's retention of key messages he learned about toothbrushing: “He has been really, I think, more aware of like where in the mouth to brush and all that since doing this which seems good.” Ava, 34 years old, rural NSW parent.

Ava's comment is similar to other parents who noted that their children's participation in the program improved their oral health literacy to improve their technique and quality of toothbrushing.

#### Attitude towards toothbrushing

The oral health program appeared to encourage children to take greater responsibility for their own oral hygiene. One mother mentioned that:

*I think he got more enthusiastic about brushing teeth himself after reading a little different bits*, *because up until then it was* ‘*momma do it*, *dadda do it’ whereas he was a bit more inclined to have a go himself*. Emma, 44 years old, metropolitan NSW parent.

Emma's testimonial reflected parents' views of how children who participated in the program at school were more enthusiastic about brushing their teeth independently at home.

Another mother commented that:

*I think the last thing I was saying was just that she is like she was okay at brushing her teeth already*, *but she was enjoying being a bit more independent*, *doing it herself*, *kind of after looking at all the pictures and after we just talked about it*. Mia, 38 years old, metropolitan NSW parent.

Mia and Emma captured parents' sentiments that their children were more enthusiastic about independently brushing their teeth because of participating in the program.

#### Oral health routine

Mothers stated that incorporating toothbrushing into a daily routine can help make it a habit for children through oral health education at school and home. One example from a mother was:

*I think a lot of it is just making it part of the routine*, *you know*, *it*'*s like you you get dressed*, *you put your shoes on*, *you brush your teeth*. *It just becomes second nature*. *And it*'*s yeah*, *it*'*s like*, *you know you*'*re not thinking about*, *you*'*re just doing it because we definitely thought that with our night‐time routine*, *the kids will actually remind us*. *It*'*s like come on now it*'*s bedtime*, *but we haven*'*t brushed our teeth*. *And then it*'*s like ah*, *okay*. *This is really good*. Emma, 44 years old, metropolitan NSW parent.

Emma's feedback aligns with other parents who mentioned that oral health becomes more of an automatic practice, and their children are more likely to remember to do it without being reminded once they develop daily routines.

In addition, another mother suggested that: “I think making it fun and engaging to engage kids and a chore that they have to do that. Turning it into something fun.” Jill, 36 years old, metropolitan NSW parent.

Jill's comment reiterated other parents' perspective that their children were more proactive in brushing their teeth once it was made more engaging and fun.

#### Oral health promotion in the family

Incorporating educational materials for parents into the children's take‐home activities can increase parental involvement to promote oral health in the family. A mother suggested that: “I think little things like the like that booklet that initiate discussion, like when they come home singing a little song or something like that, and that kick starts a conversation about it.” Amelia, 29 years old, rural NSW parent.

Similar to Amelia, parents proposed that bringing home educational materials related to oral health promotion, such as booklets or songs, can initiate conversations between parents and children about the topic.

Another mother explained:

*I think it was probably more beneficial that he brought it home because it was able to*, *you know*, *spark that conversation from him to go* "*oh what have you been learning type thing?*". *Yeah*, *because obviously in the newsletter things that that parents just skim over it sometimes or they don*'*t read it or that kind of stuff*. *But obviously if the children bring it home and they*'*re more active in that and obviously you*'*re going to have that conversation with them around that as well*. Charlotte, 37 years old, rural NSW parent.

Charlotte's comment highlighted that parents may not always pay close attention to newsletters or other materials sent to them. However, when children bring something home, it can spark parents' interest and lead to more engagement and discussion about the subject.

### Strategies for enhancing the BSBF oral health program

#### Increase oral health literacy on oral health routines and dietary intake

Parents recommended that discussion of parental oral health knowledge and the passing down of oral health habits and routines, which can help to promote oral health messages. A mother mentioned:

Our personal struggle is getting teeth brushed in the morning and doing it twice a day. I struggle with that too, and that comes down to us all being ADHD families. And it's been something that was out of routine for me. I was never (taught) as a kid, my parents just (said) like brush at night. And so that was not a routine that was stuck to me. So I really struggle to keep that in my routine and as a result, pass it on to him. Ava, 34 years old, rural NSW parent.

Ava expressed several parents' view of the difficulty in creating a routine for oral health success. Further emphasizing the need to ensure that oral health literacy is taught by caregivers to enhance familial oral health.

In regard to wanting more dietary instructions in relation to oral health, another mother commented that:

*I would like* … *some quantifiable things*, *like I know that the kids shouldn*'*t have sugar yet and all that kind of stuff*, *but I feel like if I could put a chart up sounds terrible*, *I probably could do this anyway*. *But like a chart up on the wall*. *That said*, *this is how much of this today*. *Yeah*, *whatever*, *something like that*. *Because I think it just gets a bit away with you sometimes*. Elizabeth, 37 years old, metropolitan NSW parent.

A few mothers, like Elizabeth, suggested using a chart on the wall to visually track the amount of sugar intake for their children. They believed that this would make it easier to quantify how much sugar their children consume to keep track and address it.

#### Format of message delivery

The parents gave feedback to improve message format delivery of the oral health program. Regarding how to best deliver oral health information to parents, a mother commented:

*I know the printed collateral is nice*, *so it*'*s just a bit redundant now*, *but just that little reminder to look for something in another place or even quite often when there*'*s a QR code that you can scan and then it brings up some information* … *So yeah*, *for me personally*, *more of an online website kind of platform is great*. Abigail, 39 years old, metropolitan NSW parent.

Mothers like Abigail suggested to include QR codes on printouts to access online materials more easily.

Furthermore, another mother recommended:

*But yeah*, *I do look online and just like things that I can print out helps like which they can colour in or*, *or just read with them like little books we like to read rather than to watch in our house*. Ella, 34 years old, metropolitan NSW parent.

Ella's comment coincided with other mothers who wanted either a hardcopy printout to promote oral health lessons with their children, or online portals for easier and quicker access to the information.

#### Increase access to oral healthcare

Some mothers recommended that dental health access could be improved by partnering with community dentists for in‐school check‐ups, which would promote more accessible oral healthcare. One mother suggested:

*I think regular like events at school with just like check*‐*ups where they have them at school*. *That helps*. *Especially when you*'*re kind of a busy parent and you can*'*t or you forget to take them to the dentist or they don*'*t have any time*. Ella, 34 years old, metropolitan NSW parent.

Parents like Ella recommended that schools should have oral health check‐ups for children. This can ease the load on busy parents to promote dental health access for children.

In relation to cost and accessibility of oral healthcare, another mother commented:

*Seeing a dentist is pretty expensive*, *like going getting in to see dentists is pretty expensive*. *If you need anything done*, *it*'*s even more expensive and it becomes quite prohibitive for a lot of people*, *which is something you guys are all over*. Maya, 39 years old, rural NSW parent.

Maya's comment reflected other parents' issues with healthcare access due to geographic location. These recommendations were more often mentioned by mothers that had children enrolled in rural NSW schools.

#### Mobile phone applications

Mothers recommended oral health delivery through phone applications (apps.) for easier access and reinforcement of family oral health routines. A mother commented how her family utilized a phone app for oral health by explaining:

*Anyway*, *we found this resource that is like an app where the kids get a Pokémon thing on their head and* … *it shows them which parts of their teeth to brush and as they brush it uncovers these little Pokémon in the middle*. *Yeah*, *it*'*s gamified*, *and*, *it has completely changed brushing teeth in our house*. *It is now* ‘*Can I brush my teeth*?’ Elizabeth, 37 years old, metropolitan NSW parent.

Elizabeth's comment highlighted other parents' views on the importance of gamification methods to promote toothbrushing and other oral health routines within the family.

Another mother commented how her family used an interactive app by describing that:

… *we used an app at one stage*, *which was quite*, *it was kind of interactive*. *It was kind of almost like a bit of a game*, *you know*, *like you brush your teeth*. *And it was*, *I feel like it was like for the duration of a song or a little jingle or something*. *And it kind of went around like*, *you know*, *front back sides all the different areas*. *Yeah*. *And I feel like* [the children] *responded well to that*. Mia, 38 years old, metropolitan NSW parent.

Mia's comment reflected other parents' recommendations on how the use of gamification and technology can make brushing teeth more engaging and enjoyable for children. Both statements emphasize how interactive technology, such as gamified brushing with interactive cartoon characters or jingles, can promote toothbrushing in the household.

## DISCUSSION

The findings provide insights to implement an oral health program in ECEC settings. To our knowledge, this study is the first study to investigate parental perspectives regarding the facilitators and barriers of implementing an oral health program for their children within early childhood centers, encompassing both metropolitan and rural regions of NSW, Australia, with a focus on ECC and an in‐the‐classroom implemented oral health program. The findings highlighted parents' varied perceptions of the oral health program, and their recommendations to use partnerships and technology to improve the program's delivery.

Mothers highlighted that encouragement of, and helpful instructions for, toothbrushing were the main facilitators of the oral health program. Mothers' impressions of oral health programs are important to understand the implementation of the BSBF oral health program and promote further oral health programs in early childhood centers. Finlayson et al.'s qualitative study that examined oral hygiene practices in children found that children become more independent over time, and one of the predominant driving factors influencing children's oral routines are their mother [39]. By encouraging their children to brush their teeth with useful instructions and guidance, mothers can empower children to develop independence and cultivate oral hygiene habits, especially in relation to toothbrushing.

This study's participants highlighted the value of the BSBF toothbrushing chart as an empowering tool that fosters children's sense of independence and adherence to an oral routine. This reflects the facilitative role of the chart and helpful instructions in enhancing the effectiveness of the oral health program. These findings correspond to previous research that found providing education to parents effectively improved their supervision of children's tooth brushing skills and overall dental hygiene routine [40]. Furthermore, the inclusion of helpful instructions and where to brush in overlooked areas such as the tongue expanded the scope of the program.

This study's findings emphasized challenges mothers and their children experienced with take‐home program materials, ECEC center attendance, and communication. These findings reflect that the perceived usefulness of brochures for parents is contingent on the provision of verbal instructions [41]. It aligns with Chandio et al. [19] in underscoring the multifaceted nature of factors influencing oral health within the home, notably emphasizing that parents' limited knowledge of teachers' role in teaching oral health is just one aspect. Social determinants, among other factors, play a significant role in shaping oral health outcomes in the family setting. Furthermore, the absence of effective communication from child to parent and inconsistent attendance at ECEC centers exacerbate the challenge by fostering ambiguity in messaging and materials within the program. This highlights the importance of delivering information to the entire family for enhancing parents' effects on oral health programs [39]. The challenges illustrated in this study highlight the necessity of in‐school oral health programs to emphasize consistent communication, monitoring children's program attendance, and provision of materials by educators to ensure a smooth transition from the classroom to the home [42].

The study reviewed the online resources for parents, such as the BSBF website for parents, which included educational infographics and videos on oral health messages. Digital resources like videos and phone applications aid in encouraging oral health knowledge, education, and promotion [43]. This was reiterated by the study's findings, which highlighted that the participants' children enjoyed the interactive elements, graphics, colors, and videos on the website. Sharma et al. [43] also highlighted that websites must optimize accessibility to ensure that information reaches parents during their spare time. Aliakbari et al. [44] recommended that online resources need to be more user‐friendly. This can accommodate parents who seek concise and time‐efficient instructions to promote oral health.

Additionally, mothers gave insight into the overall impact of the oral health program. Research has shown that parents often provide the first example of oral health routines for children, and these behaviors can be passed on generationally [45]. Toothbrushing is one of the fundamental aspects of oral hygiene that correlates directly with ECC. Kumar et al. [46] found that infrequent brushers have 1.5 times higher odds of developing new caries when comparing individuals that brushed their teeth one or more times per day. This study's findings revealed that some mothers did not know the correct locations of the mouth to brush and admitted that their children might not brush twice a day, every day. Through the oral health program, they could learn these locations and further support their children in retaining this information and practicing better oral hygiene routines.

The findings reaffirm the need to educate parents on oral hygiene and create a routine within the family to ensure proper habits in oral health [47]. Our study found that after the program, mothers observed positive changes in their child's attitude toward toothbrushing and their family's oral health routine following the program. Children showed increased enthusiasm for independent brushing, heightened awareness of brushing frequency, longer brushing durations, and improved self‐observation during brushing. It is important to note that the children within this study ranged between 2 and 5 years old, which is not regarded as the appropriate developmental age for children to fully gain independence brushing their own teeth [48]. The findings from our study give unique insights into the beginning stages of oral health routine formation in the family and the importance of parental education to support their child's learning in oral health.

Moreover, mothers recommended incorporating performative educational strategies into children's take‐home activities can increase children and parents' engagement in oral health at home. Mothers used gamification techniques such as phone apps to promote educational messages of oral health programs. Fijačko et al. [49] determined that using apps for oral education and care can promote motivation for oral hygiene in children. Furthermore, the usage of songs for health promotion is an effective method to deliver information in a low‐cost and enjoyable way [50]. Overall, oral health programs could benefit by adding in a song to tie the storyline and educational materials together for the teachers, children, and parents to practice and reinforce.

Mothers' recommendations underscore the importance of oral health programs developing partnerships with local dentists to enhance accessibility and affordability of oral healthcare services for children in rural areas [45]. This is crucial because children in rural Australia exhibit higher rates of dental caries compared with their metropolitan counterparts [42]. According to mothers in the study, establishing community partnerships through initiatives such as in‐school dental services and enhanced referral pathways [45] can facilitate free check‐ups and promote overall dental hygiene during early childhood, particularly in socioeconomically disadvantaged rural regions.

The metropolitan areas may be influenced by factors such as potentially more diverse socio‐economic backgrounds due to their higher SEIFA rankings [30]. On the other hand, rural towns according to the MMA, such as Bathurst and Cessnock could face additional challenges in delivering early childhood oral health programs due to potential geographic isolation and lower socio‐economic status, as indicated by their SEIFA rankings [30]. These disparities underscore the critical need for tailored approaches to address the unique social determinants of health prevalent in rural communities.

The current approach to pediatric oral health education in Australia involves various initiatives. This includes school and community‐based programs, policy‐driven dental services such as the Child Dental Benefits Schedule (CDBS) [51], and oral health promotional campaigns by the Australian Government [52]. However, despite these efforts, there are shortcomings in the current practice, such as insufficient parental knowledge regarding oral health, health disparities within families, limited accessibility to oral health services in rural areas, and lack of referral pathways [53]. Addressing these issues requires future research to emphasize preventive measures and foster collaboration among ECEC settings and oral health programs. Future research should prioritize preventive measures and explore in‐school programs, including toothbrushing activities and educational lessons on nutrition, to promote oral health in ECEC settings.

### Limitations

This study had limitations such as lacking the perspectives of fathers because only mothers indicated an interest to participate in the study. Since research has shown fathers' involvement to be valuable in various aspects of child health [54], fathers' perspectives should be explored in future research to contribute a more diverse parental viewpoint on oral health programs. The study's sample lacked cultural and ethnic diversity, which should be sought in future to examine whether parents' ethnocultural practices inform children's oral health. In addition, the study's sample lacked parents with varying educational levels, which may impact their perceptions and oral health literacy. As a result of having a small sample size, findings from this study may not be easily generalized to broader populations or contexts. While the use of videoconferencing platforms to conduct interviews and focus groups overcomes spatial and financial barriers to participate, it may discriminate against participants who cannot access video‐compatible technology [31].

## CONCLUSION

This qualitative study aided in identifying several facilitators and challenges faced by parents and children when implementing oral health programs. The findings highlight the need for continuous improvement in oral health programs and seeking parents' feedback for pediatric oral health. The study's findings can raise awareness among policymakers, healthcare professionals, and the public regarding the importance of implementing oral health programs for preschool‐aged children and understanding parental perceptions. This increased awareness can drive policy discussions and public health agendas. It can lead to more advocacies to attract additional funding for pediatric dental services; promote preventive measures; and foster partnerships between dental professionals, organizations, and government bodies to enhance evidence‐based policies and research initiatives. These are effective strategies for policy to enhance oral health programs and reduce ECC among the vulnerable group of young children.

## FUNDING INFORMATION

The study was funded by the School of Health Sciences (Western Sydney University) Collaborative Multidisciplinary Partnership Grant in collaboration with Colgate Palmolive Pty. Ltd.

## CONFLICT OF INTEREST STATEMENT

The authors declare no conflicts of interest.

## Supporting information


**Table S1.** Criteria for reporting qualitative research checklist.

## Data Availability

The data presented in this study are not publicly available due to privacy. Further information pertaining to data will be available upon reasonable request from the corresponding author's email address.
